# Real-World Effectiveness of Intravenous and Oral Antibiotic Stepdown Strategies for Gram-Negative Complicated Urinary Tract Infection With Bacteremia

**DOI:** 10.1093/ofid/ofae193

**Published:** 2024-04-04

**Authors:** John J Veillette, Stephanie S May, Sameer Alzaidi, Jared Olson, Allison M Butler, C Dustin Waters, Katarina Jackson, Mary A Hutton, Brandon J Webb

**Affiliations:** Infectious Diseases Telehealth Service, Intermountain Health, Murray, Utah, USA; Department of Pharmacy, Intermountain Medical Center, Murray, Utah, USA; Infectious Diseases Telehealth Service, Intermountain Health, Murray, Utah, USA; Department of Pharmacy, Intermountain Medical Center, Murray, Utah, USA; Pharmacy Services, Intermountain Health, Taylorsville, Utah, USA; Department of Pharmacy, Primary Children's Hospital, Salt Lake City, Utah, USA; Division of Pediatrics, University of Utah, Salt Lake City, Utah, USA; Statistical Data Center, Intermountain Health, Murray, Utah, USA; Department of Pharmacy, McKay-Dee Hospital, Ogden, Utah, USA; Department of Pharmacy, Intermountain Medical Center, Murray, Utah, USA; Department of Pharmacy, Utah Valley Hospital, Provo, Utah, USA; Division of Clinical Epidemiology and Infectious Diseases, Intermountain Medical Center, Murray, Utah, USA

**Keywords:** antimicrobial stewardship, β-lactams, complicated urinary tract infection, gram-negative bacteremia, real-world evidence

## Abstract

**Background:**

Robust data are lacking regarding the optimal route, duration, and antibiotic choice for gram-negative bloodstream infection from a complicated urinary tract infection source (GN-BSI/cUTI).

**Methods:**

In this multicenter observational cohort study, we simulated a 4-arm registry trial using a causal inference method to compare effectiveness of the following regimens for GN-BSI/cUTI: complete course of an intravenous β-lactam (IVBL) or oral stepdown therapy within 7 days using fluoroquinolones (FQs), trimethoprim-sulfamethoxazole (TMP-SMX), or high-bioavailability β-lactams (HBBLs). Adults treated between January 2016 and December 2022 for *Escherichia coli* or *Klebsiella* species GN-BSI/cUTI were included. Propensity weighting was used to balance characteristics between groups. The 60-day recurrence was compared using a multinomial Cox proportional hazards model with probability of treatment weighting.

**Results:**

Of 2571 patients screened, 759 (30%) were included. Characteristics were similar between groups. Compared with IVBLs, we did not observe a difference in effectiveness for FQs (adjusted hazard ratio, 1.09 [95% confidence interval, .49–2.43]) or TMP-SMX (1.44 [.54–3.87]), and the effectiveness of TMP-SMX/FQ appeared to be optimal at durations of >10 days. HBBLs were associated with nearly 4-fold higher risk of recurrence (adjusted hazard ratio, 3.83 [95% confidence interval, 1.76–8.33]), which was not mitigated by longer treatment durations. Most HBBLs (67%) were not optimally dosed for bacteremia. Results were robust to multiple sensitivity analyses.

**Conclusions:**

These real-world data suggest that oral stepdown therapy with FQs or TMP-SMX have similar effectiveness as IVBLs. HBBLs were associated with higher recurrence rates, but dosing was suboptimal. Further data are needed to define optimal dosing and duration to mitigate treatment failures.

Oral antibiotic stepdown therapy is a safe, convenient, less costly alternative to outpatient intravenous antibiotics for gram-negative bloodstream infections (GN-BSIs) [1–5]. However, data are lacking regarding the optimal oral agent, dose, and duration for complicated urinary tract infection (cUTI), which suffers from variable definitions in published literature and (when defined as structural or functional urologic abnormalities) often comprises a minority of included patients in GN-BSI studies [6–15]. Randomized trials have found a duration of 7 days to be sufficient for afebrile cUTI treated with a fluoroquinolone (FQ) or trimethoprim-sulfamethoxazole (TMP-SMX), whereas >7 days may be needed for febrile cUTI (depending on the chosen definition of treatment success) [16, 17]. Recent observational studies suggest that 7–10 days may be appropriate for GN-BSI from a cUTI source (GN-BSI/cUTI) [18] and that oral β-lactams (BLs) might be associated with slightly higher recurrence rates of questionable clinical significance [19].

Many of these studies were limited by small sample size, variable definitions of treatment failure, and underrepresentation or underdosing of oral BLs. Further data are needed to optimize oral stepdown therapy for GN-BSI/cUTI. Herein, we present real-world data from a large, observational, multicenter cohort study using a target trial emulation with a causal inference method to compare effectiveness between IVBLs and oral stepdown with BLs, FQs, or TMP-SMX for GN-BSI/cUTI.

## METHODS

### Patients and Data Collection

The study took place in the Intermountain Health integrated network of 23 hospitals and emergency departments (EDs), 38 urgent cares, and 300 primary care clinics in Utah and Idaho, serving >1.5 million patients each year. Using the enterprise data warehouse, we identified a screening population of unique patients ≥18 years of age with matching positive blood and urine cultures for *Escherichia coli*, *Klebsiella pneumoniae*, or *Klebsiella oxytoca* obtained during an ED or hospital encounter between January 2016 and December 2022.

Data related to hospital course, demographics, laboratory values, and microbiology were extracted electronically from the enterprise data warehouse, whereas data on comorbid conditions, imaging, severity of illness, antibiotic treatment, recurrent infection, readmissions, and deaths were abstracted manually from the electronic medical record (EMR) by trained record reviewers using a standardized data collection tool (see Appendix in the Supplementary Materials). Data were collected through 90 days after hospital discharge. The study met all STROBE (Strengthening the Reporting of Observational Studies in Epidemiology) requirements for observational studies [20] and ISPOR criteria for comparative effectiveness [21], and it was approved by the Intermountain Institutional Review Board; it was granted a waiver of patient consent due to its design and less-than-minimal risk to subjects.

### Inclusion and Exclusion Criteria

Male and female patients with cUTI, defined by the presence of structural or functional urologic abnormalities, were included in the initial screening cohort. We excluded patients who had concomitant infections during the index encounter (besides GN-BSI/cUTI), polymicrobial cultures, hospital-onset bacteremia, or a nonurinary source of bacteremia (eg, prostatitis, epididymo-orchitis, or renal abscess). Furthermore, we excluded patients who were pregnant, died in the hospital, were discharged with hospice, transferred to a facility outside our healthcare system, or were lost to follow-up after discharge (defined as no further notes or follow-up visits in the record). Patients were also excluded if they had a prolonged hospitalization (>14 days), did not receive an effective intravenous antibiotic within 24 hours of the index blood culture, received >7 days of intravenous antibiotics before discharge or oral stepdown, received multiple oral antibiotics, or received an oral antibiotic to which the blood or urine isolate was not susceptible or if EMR data were incomplete regarding antibiotic treatment.

### Microbiology Procedures

Blood culture susceptibilities were performed on either BD Phoenix (BD Diagnostic Systems) or MicroScan Walkaway (Beckman Coulter) panels depending on the processing facility, whereas all urine cultures were processed on MicroScan Walkaway panels. Granular minimum inhibitory concentration (MIC) data were lacking for many isolates reported only as “susceptible” in our EMR, and we often had to infer susceptibility for oral antibiotics (eg, cephalexin and amoxicillin) based on surrogate intravenous antibiotics reported on the panel (eg, cefazolin and ampicillin, respectively). Because of this limitation, we preplanned an analysis limited to isolates confirmed to be susceptible by current Clinical and Laboratory Standards Institute (CLSI) breakpoints (see Sensitivity Analyses section).

### Enrollment Window and Index Day 0

Index day 0 for the simulated trial was defined as the index blood culture date. The enrollment window was defined as index day 1 through day 7 to reflect real-world variation in the time to culture positivity and clinical stability (ie, afebrile and hemodynamically stable). Patients were permitted to transition from intravenous to oral antibiotics at any time during the enrollment window per the treating physician's discretion, provided that they were transitioned to oral stepdown therapy or discharged on IVBL therapy by index day 7.

### Comparator Groups

Patients were classified into 1 of 4 comparator groups based on the definitive treatment they received: intravenous BL (IVBL), oral FQ (levofloxacin and ciprofloxacin), oral TMP-SMX, or high-bioavailability oral BLs (HBBLs; amoxicillin, amoxicillin-clavulanate, and cephalexin). For sensitivity analyses, we also evaluated a fifth group receiving low-bioavailability oral BLs (LBBLs; cefdinir and cefuroxime). IVBLs were used as the reference group in all comparisons. Oral antibiotic dosing and duration was per prescriber choice in this real-world study. However, we prespecified a descriptive analysis of patients who received bacteremia dosing per Delphi expert consensus recommendations [10] (with appropriate adjustments for renal impairment; all taken orally): ciprofloxacin (750 mg every 12 hours), levofloxacin (750 mg every 24 hours), TMP-SMX (5 mg/kg every 12 hours; eg, approximately 2 double-strength tablets every 12 hours for a 70-kg patient), amoxicillin (1000 mg every 8 hours), amoxicillin/clavulanic acid (875–1000 mg every 8 hours), and cephalexin (1000 mg every 6 hours).

### Outcomes and Primary Analysis

The primary outcome was recurrence-free days through index day 60. Recurrence was defined as positive blood or urine culture for the same organism (regardless of susceptibility results). If only the urine culture was positive (ie, no evidence of recurrent bacteremia), manual review of the EMR had to indicate that a symptomatic urinary tract infection (UTI) was diagnosed and treated with antibiotics in order to count as a recurrence. Otherwise, positive urine cultures without documented symptoms and treatment were classified as asymptomatic bacteriuria and were not counted toward the primary outcome. Secondary outcomes included all-cause mortality, *Clostridioides difficile* infection, and UTI-related readmission rates. All ED or hospital readmissions were manually reviewed to determine whether they were UTI related, defined as either an adverse event to the antibiotic or ongoing, worsening, or recurrent UTI as the reason for readmission. Adjudication of all patients with a primary or secondary outcome was performed by the same infectious diseases pharmacist (J. J. V.) to ensure consistency.

### Statistical Analysis

To address exchangeability, we used a 4-comparator multinomial regression model to estimate propensity weights for choosing one antibiotic group over the others (R; twang package) [22–25]. The propensity model was fitted using covariates identified from relevant GN-BSI literature [2, 18] and causal diagrams: age, sex, chronic kidney disease, Charlson comorbidity index, Pitt bacteremia score, allergy or resistance to FQs/TMP-SMX/BLs, and small hospital size (<200 beds; due to observed variation in prescribing patterns between large and small hospitals in our health system). We selected an optimally balanced model for the primary analysis based on the absolute standardized mean differences, minimum *P* values, and effective sample sizes (Supplementary Table 1). Furthermore, we assessed covariate overlap via propensity score box plots, which, together with the balance table, supported causal estimation (Supplementary Figure 1). We then compared recurrence risk between groups using a propensity score–weighted multinomial Cox proportional hazards model. The model was censored at the time of death, administration of antibiotics during a subsequent ED/hospital admission (for indications other than GN-BSI/UTI), or last known follow-up within 90 days (rather than prespecifying separate per-protocol and intention-to-treat analyses). Duration of intravenous antibiotic treatment, in days, was included as a time-dependent covariate in the model because the enrollment window allowed up to 7 days of intravenous antibiotic treatment before oral switch.

Regarding antibiotic treatment duration, in a slight divergence from a strict trial emulation method (whereby duration subgroups would ideally be stratified at enrollment), we chose to optimize power and recognize uncertainty in the existing evidence [11, 18, 26] by including total antibiotic duration as a continuous, time-dependent variable in the primary analysis. We then conducted prespecified analyses to explore associations between recurrence and total duration. To determine an optimal cutoff point for dichotomizing duration, we used a receiver operating characteristic curve to plot 60-day recurrence versus antibiotic duration and calculated the Youden index [27] that optimized sensitivity and specificity for all antibiotics. We then refit the primary Cox models, using the duration cutoff point as an interaction term, to estimate the effect of each oral antibiotic on recurrence stratified by short versus long duration. Results were displayed by creating cumulative incidence plots.

### Sensitivity Analyses

Several sensitivity analyses were planned a priori. First, we evaluated the primary analysis for recurrence at index days 30 and 90. Second, we expanded the BL group in the primary analysis to include all BLs (LBBLs and HBBLs together). Third, we limited the BL group in the primary analysis to only those patients receiving HBBLs whose blood and urine isolates were confirmed to be susceptible at current CLSI breakpoints (cefazolin ≤MIC 2 mg/L if they received cephalexin, ampicillin MIC ≤8 mg/L if they received amoxicillin, or amoxicillin-clavulanate MIC ≤8/4 mg/L if they received amoxicillin-clavulanate) [28]. Fourth, we conducted a variation of the primary analysis with restricted inclusion criteria allowing 1–4 days (instead of 1–7 days) of intravenous antibiotics before oral switch. Finally, we fitted a new 3-group Cox model to compare LBBLs, HBBLs, and IVBLs.

## RESULTS

**Figure 1.**
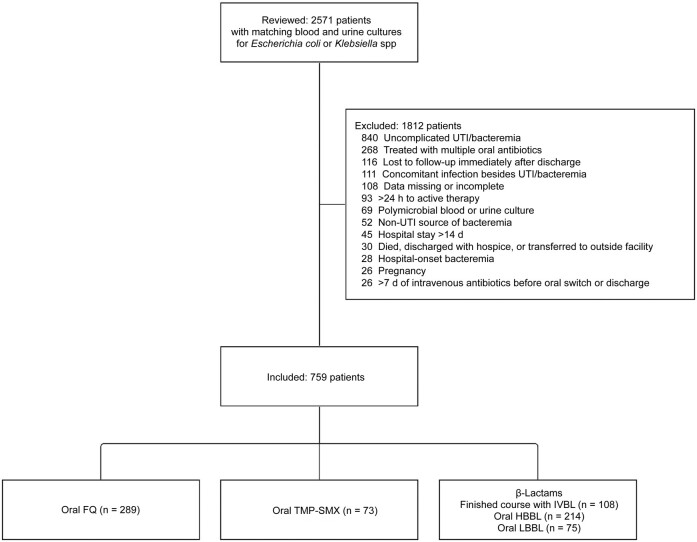
Patient inclusion/exclusion. Abbreviations: FQ, fluoroquinolone; HBBL, high-bioavailability β-lactam; IVBL, intravenous β-lactam; LBBL, low-bioavailability β-lactam; TMP-SMX, trimethoprim-sulfamethoxazole; UTI, urinary tract infection.

Of 2571 patients reviewed, 759 (30%) met inclusion criteria (Figure 1). There were 289 patients in the FQ group, 73 in the TMP-SMX group, 214 in the HBBL group, and 108 in the IVBL group. Most patients (713 [94%]) were treated in the hospital, and 190 (25%) required intensive care (Table 1). Patients were predominantly female (470 patients [62%]) and >65 years of age (461 [61%]). Baseline characteristics were similar across the oral stepdown groups, except that patients receiving TMP-SMX had the highest incidence of urinary retention, kidney stones, and hydronephrosis (26%, 53%, and 55%, respectively). Patients receiving definitive IVBLs had more comorbid conditions, urologic abnormalities, and extended-spectrum β-lactamase­–producing isolates than the oral stepdown groups. Most patients (702 [92%]) achieved clinical stability within the first 3 days, and nearly all (751 [99%]) were initially treated with an IVBL before oral stepdown or discharge on definitive IVBL therapy. The median time to oral switch (interquartile range) was 3 (3–4) days, and the median total duration of antibiotic treatment was 14 (11–15) days. Oral antibiotic prescribing patterns varied by care venue: ED patients were most likely to receive an HBBL (22 of 46 [48%]), hospitalized patients were most likely to receive an FQ (278 of 713 [39%]), and patients transitioned to TMP-SMX more frequently received care at smaller hospitals (38 of 73 [52%]). More patients treated with FQs received consensus-recommended dosing compared with those treated with TMP-SMX (69% vs 4%; *P* < .001) or HBBLs (69% vs 43%; *P* < .001) (Table 1).

**Table 1.** Demographics, Comorbid Conditions, and Antibiotic Treatment

Baseline Characteristics	Patients, No. (%)^a^				
	FQ (n = 289)	TMP-SMX (n = 73)	HBBL (n = 214)	IVBL (n = 108)	LBBL^b^ (n = 75)
Age, median (IQR), y	67 (55–76)	68 (56–78)	73 (61–81)	72 (61–79)	71 (60–77)
Female sex	188 (65)	37 (51)	135 (63)	72 (67)	38 (51)
Diabetes	138 (48)	27 (37)	104 (49)	54 (50)	31 (41)
Chronic kidney disease (stage II or higher)	80 (28)	19 (26)	80 (37)	48 (44)	23 (31)
Charlson comorbidity index, median (IQR)	6 (3–9)	4 (2–8)	6 (4–9)	7 (5–10)	5 (3–8)
Allergy to FQ, TMP-SMX, or BL	18 (6)	2 (3)	4 (2)	13 (12)	12 (16)
History of kidney stones	107 (37)	35 (48)	51 (24)	31 (29)	26 (35)
Immunocompromised^c^	47 (16)	5 (7)	47 (22)	41 (38)	13 (17)
Urinary retention or neurogenic bladder	55 (19)	19 (26)	39 (18)	25 (23)	10 (13)
Benign prostatic hypertrophy	51 (18)	18 (25)	35 (16)	16 (15)	16 (21)
Baseline urinary catheterization	28 (10)	14 (19)	27 (13)	21 (19)	7 (9)
Chronic urinary incontinence	30 (10)	4 (5)	33 (15)	12 (11)	8 (11)
Urologic stricture, stenosis, or obstruction	35 (12)	7 (10)	20 (9)	13 (12)	8 (11)
Cancer or mass of bladder, prostate, or kidney	22 (8)	7 (10)	27 (13)	7 (6)	5 (7)
>2 Positive urine cultures in past year	13 (4)	6 (8)	10 (5)	11 (10)	4 (5)
Urologic procedure in previous 2 wk	12 (4)	3 (4)	8 (4)	8 (7)	3 (4)
Baseline stent or nephrostomy tube	10 (3)	3 (4)	9 (4)	7 (6)	1 (1)
Cystocele or urologic fistula	6 (2)	2 (3)	7 (3)	3 (3)	2 (3)
Other urologic abnormalities^d^	7 (2)	0 (0)	4 (2)	2 (2)	2 (3)
Index bacteremia characteristics					
Active kidney/ureteral stone	130 (45)	39 (53)	69 (32)	30 (28)	27 (36)
Hydronephrosis or hydroureter	106 (37)	40 (55)	63 (29)	35 (32)	26 (35)
Ureteral stent or nephrostomy tube placed	75 (26)	26 (36)	41 (19)	25 (23)	23 (31)
Received care at small hospital (<200 beds)	105 (36)	38 (52)	88 (41)	36 (33)	34 (45)
Admitted to hospital	278 (96)	69 (95)	192 (90)	105 (97)	69 (92)
Discharged from emergency department	11 (4)	4 (5)	22 (10)	3 (3)	6 (8)
Length of stay, median (IQR), h	69 (48–87)	71 (51–91)	72 (49–96)	91 (56–108)	64 (36–87)
Pitt bacteremia score, median (IQR)	2 (1–3)	3 (1–3)	2 (1–3)	2 (1–3)	2 (1–3)
Admitted to intensive care unit	50 (17)	20 (27)	58 (27)	40 (37)	22 (29)
Received vasopressors	17 (6)	15 (21)	31 (14)	19 (18)	15 (20)
Achieved clinical stability within 3 d	269 (93)	63 (86)	203 (95)	100 (93)	67 (89)
Microbiology					
*Escherichia coli*	234 (81)	61 (84)	175 (82)	88 (81)	64 (85)
*Klebsiella* species	55 (19)	12 (16)	39 (18)	20 (19)	11 (15)
FQ resistant	0 (0)	13 (18)	31 (14)	50 (46)	14 (19)
TMP-SMX resistant	52 (18)	0 (0)	47 (22)	43 (40)	20 (27)
Cefazolin resistant	33 (11)	10 (14)	3 (1)	40 (37)	7 (9)
ESBL-producing isolate	4 (1)	1 (1)	0 (0)	23 (21)	0 (0)
Antibiotic treatment					
Empiric IVBL	283 (98)	73 (100)	212 (99)	108 (100)	75 (100)
Empiric intravenous FQ	6 (2)	0 (0)	2 (1)	0 (0)	0 (0)
Time to active intravenous therapy, median (IQR), h	1.1 (0.6–1.8)	0.7 (0.3–1.8)	1.2 (0.5–1.8)	1.6 (0.7–2.2)	1.2 (0.4–1.9)
Duration of active inpatient intravenous therapy, median (IQR), d	3 (3–4)	4 (3–5)	4 (3–5)	4 (3–5)	3 (2–4)
Duration of active oral therapy, median (IQR), d	10 (7–12)	10 (7–11)	10 (7–11)	NA	10 (7–11)
Total duration, median (IQR), d	13 (10–15)	13 (11–15)	13 (11–15)	15 (13–16)	13 (11–15)
Received recommended oral dosing^e^	199 (69)	3 (4)	93 (43)	NA	NA^f^

Abbreviations: BL, β-lactam; ESBL, extended-spectrum β-lactamase, FQ, fluoroquinolone, HBBL, high-bioavailability BL; IQR, interquartile range; IVBL, intravenous BL; LBBL, low-bioavailability BL; NA, not applicable; TMP-SMX, trimethoprim-sulfamethoxazole.

^a^Data represent no. (%) of patients unless otherwise specified.

^b^The LBBL group was included in sensitivity analyses but not in the primary analysis.

^c^Immunocompromised was defined as follows: human immunodeficiency virus/AIDS with a CD4 cell count <200/µL, neutropenia with an absolute neutrophil count <500/µL, or receiving any of the following medications at the time of admission: antirejection medications following transplant, chemotherapy, tumor necrosis factor α inhibitors, disease-modifying antirheumatic drugs, or maintenance steroids with equivalent prednisone dose ≥20 mg.

^d^Other urologic abnormalities included urostomy (n = 9), neobladder (n = 2), bladder sling (n = 1), polycystic kidney disease (n = 1), solitary kidney (n = 1), and chronic interstitial cystitis (n = 1).

^e^Recommended oral antibiotic doses were based on consensus guidance from Heil et al [10].

^f^LBBLs are not routinely recommended for GN-BSI due to pharmacokinetic concerns and lack of clinical data.

Crude 60-day recurrence occurred in 111 patients (14.6%) and was lowest (overall and when considering bacteremia and UTI separately) in those receiving FQ or TMP-SMX stepdown (Table 2). Recurrence was primarily driven by recurrent UTIs (12.0% vs 2.6% for recurrent bacteremia + UTI). Unadjusted all-cause 90-day mortality and *C difficile* infection rates were low overall (1.7%, and 1.2%, respectively) and did not differ significantly between groups.

**Table 2.** Unadjusted Primary and Secondary Outcomes

Outcome	Patients, No. (%)				
	FQ (n = 289)	TMP-SMX (n = 73)	HBBL (n = 214)	IVBL (n = 108)	LBBL (n = 75)
Primary outcomes					
Recurrence within 60 d	21 (7.3)	7 (9.6)	46 (21.5)	22 (20.4)	15 (20.0)
Recurrent bacteremia + UTI	3 (1.0)	1 (1.4)	9 (4.2)	5 (4.6)	2 (2.7)
Recurrent UTI only	18 (6.2)	6 (8.2)	37 (17.3)	17 (15.7)	13 (17.3)
Secondary outcomes					
Recurrence within 30 d	12 (4.2)	4 (5.5)	31 (14.5)	15 (13.9)	10 (13.3)
Recurrence within 90 d	26 (9.0)	11 (15.1)	56 (26.2)	32 (29.6)	17 (22.7)
UTI-related readmission within 90 d	28 (9.7)	11 (15.1)	46 (21.5)	35 (32.4)	13 (17.3)
*Clostridioides difficile* infection within 90 d	0 (0.0)	1 (1.4)	3 (1.4)	4 (3.7)	1 (1.3)
All-cause 90-d mortality rate	3 (1.0)	1 (1.4)	5 (2.3)	2 (1.9)	2 (2.7)

Abbreviations: FQ, fluoroquinolone; HBBL, high-bioavailability β-lactam; IVBL, intravenous β-lactam; LBBL, low-bioavailability β-lactam; TMP-SMX, trimethoprim-sulfamethoxazole; UTI, urinary tract infection.

**Figure 2.**
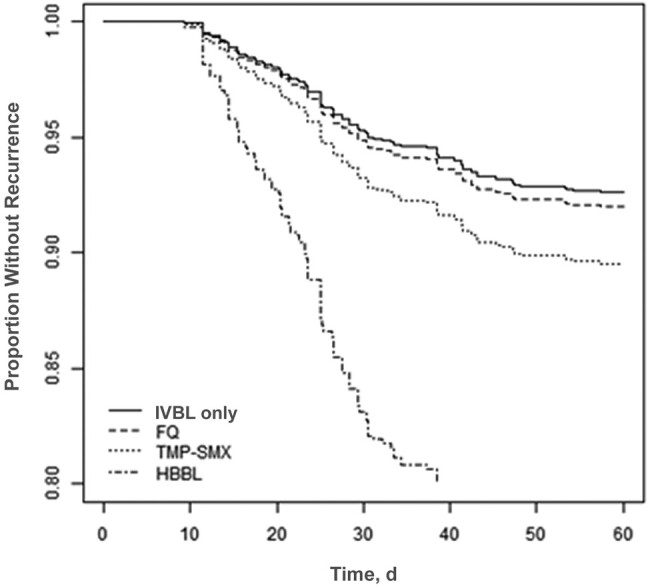
Recurrence-free days for gram-negative bloodstream infection from a complicated urinary tract infection source, comparing intravenous β-lactam (IVBL) versus oral stepdown therapies. Data represent cumulative incidence curves generated from the propensity-weighted Cox proportional hazards models. Abbreviations: FQ, fluoroquinolone; HBBL, high-bioavailability β-lactam; TMP-SMX, trimethoprim-sulfamethoxazole (all taken orally).

**Figure 3.**
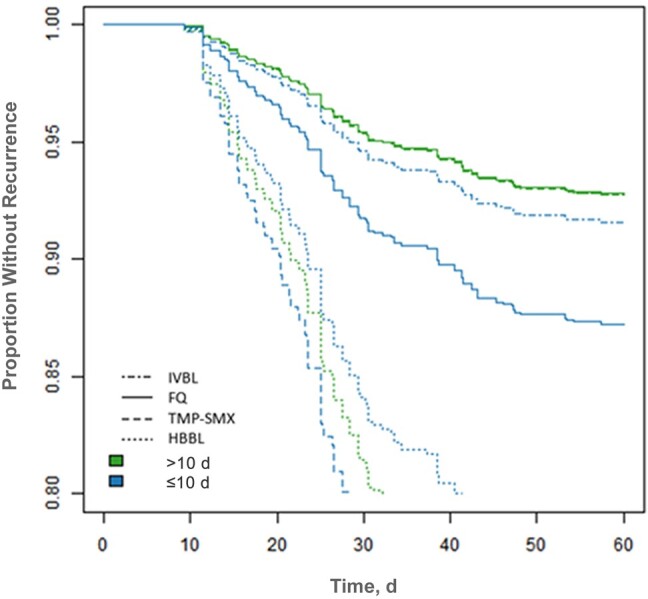
Recurrence-free days through day 60 for gram-negative bloodstream infection from a complicated urinary tract infection source, based on treatment regimen and total duration. Data represent cumulative incidence curves generated from the propensity-weighted Cox proportional hazards models. Abbreviations: FQ, fluoroquinolone, HBBL, high-bioavailability β-lactam; IVBL, intravenous β-lactam; TMP-SMX, trimethoprim-sulfamethoxazole (all taken orally).

In the primary analysis, compared with a full course of IVBL, we did not observe a difference in recurrence with oral stepdown to FQs (adjusted hazard ratio [aHR], 1.09 [95% confidence interval (CI), .49–2.43]) or TMP-SMX (aHR 1.44 [.54–3.87]). However, the recurrence risk was nearly 4-fold higher with HBBLs (aHR, 3.83 [95% CI, 1.76–8.33]; *P* < .001) (Figure 2). Receiver operating characteristic curve analysis identified 10 days as the best cutoff point in our data set for differentiating the effect of total antibiotic duration on 60-day recurrence, although there were not large differences in discrimination using other cutoff points (Supplementary Figure 2). In the modified primary analysis model, each antibiotic group was stratified by short (≤10 days) versus longer (>10 days) total duration. Compared with “longer-duration IVBL” as the referent group, we did not observe significant differences in recurrence risk for short-duration IVBL (aHR, 1.17 [95% CI, .29–4.74]), longer duration with FQ stepdown (0.99 [.38–2.58]), or longer duration with TMP-SMX stepdown (1.00 [.29–3.49]). However, longer duration with HBBL stepdown (aHR, 4.35 [1.78–10.66]), short duration with HBBL stepdown (3.68 [1.36–9.91]), and short duration with TMP-SMX stepdown (5.28 [1.36–20.46]) were all associated with significantly higher recurrence risk (Figure 3). Short duration with FQ stepdown was associated with an aHR of 1.82 (.64–5.23), which was not significantly different, but this analysis was limited by small sample size.

Results were robust to all sensitivity analyses (Supplementary Figures 3–7). We did not detect a difference in recurrence risk between HBBLs and LBBLs, both of which were associated with significantly higher recurrence than IVBL (Supplementary Figure 8). Given concerns with low HBBL dosing in our study, we conducted a limited post hoc subgroup analysis of dosing and 60-day recurrence stratified by renal function and recurrence stratified by dosing (using unadjusted data). This suggested that preserved renal function (Supplementary Table 2) and suboptimal dosing (Supplementary Table 3) might both contribute to HBBL recurrence, although this analysis was limited by small sample size.

## DISCUSSION

Our real-world data provide several important insights on treatment of GN-BSI/cUTI. First, we observed no difference in effectiveness between a full IVBL course and oral stepdown to FQs or TMP-SMX. This finding was most notable for the TMP-SMX group, which had more patients with urologic abnormalities and fewer patients receiving recommended dosing [10] than the FQ group. Together with other published data [6, 9, 12, 18, 19], our findings suggest that oral stepdown with FQ or TMP-SMX should be preferred for GN-BSI/cUTI once clinical stability is achieved on intravenous therapy.

Second, FQ and TMP-SMX stepdown appeared to be optimized with total durations longer than 10 days. While 2 randomized trials [7, 11] have found comparable outcomes with shorter (ie, 7-day) durations for GN-BSI, it is important to note that both trials excluded patients with lack of source control (often a concern for patients with cUTI), and one specifically excluded patients with moderate to severe hydronephrosis [11] (which was prevalent in our data set). It is possible that 7-day courses for GN-BSI from a UTI source are most effective when the patient either lacks structural or functional urologic abnormalities or has source control. Of note, we observed no significant difference in recurrence risk for short versus long duration with FQ stepdown, but the recurrence risk for short-course TMP-SMX stepdown was strikingly higher. These findings, particularly for TMP-SMX, raise the question of how best to optimize dosing and duration for effectiveness while limiting toxicity. Further study is needed to clarify whether interventions such as higher dosing combined with shorter duration could optimize outcomes for TMP-SMX stepdown therapy.

Finally, oral stepdown with HBBLs was associated with the highest risk of recurrence. While some studies have not observed higher recurrences with HBBLs [8, 13, 14], this finding is consistent with a previous meta-analysis and multicenter VA cohort study [9, 19]. HBBL recurrences were not mitigated by longer treatment duration in our data set, but this analysis was limited by small sample size and suboptimal dosing. It is possible that optimally dosing HBBL or prolonging treatment durations (>10 days) might close the outcomes gap in patients with GN-BSI/cUTI, but further study is needed.

Our study adds robust, real-world evidence to a growing knowledge base and had many advantages, including large sampling of only patients with cUTI, granular data from manual record review, use of a causal inference method, capturing recurrent symptomatic UTI in addition to bacteremia, and prolonged 90-day surveillance in an integrated health system. However, we acknowledge some important limitations, including unmeasured confounders that may have influenced prescribing choices and incomplete data that may have contributed to recurrence risk (eg, patient compliance, postdischarge change in antibiotics, or bacterial virulence). We were unable to control for provider-level or facility-level variability. The timing of postdischarge urologic procedures for source control and restoration of urinary flow (eg, lithotripsy with ureteral stent removal) might have influenced recurrence in any of the treatment groups; however, we could not accurately capture this information because many procedures took place outside our healthcare system. Readmissions and recurrences outside our system were not captured, which may have led to underreporting of outcomes. We could not distinguish between recurrence and new infection during retrospective review; however, sensitivity analyses at days 30, 60, and 90 yielded similar results.

Our restrictive inclusion criteria limited the study patients to only 30% of the screened population, which limits external validity, and our conclusions may apply only to patients who achieve clinical stability within 3 days on intravenous therapy. Furthermore, we acknowledge that “cUTI” represents a heterogeneous group of patients, and our findings might apply differently to various subgroups. Finally, our ability to evaluate the effectiveness of HBBLs was limited by current susceptibility testing practices, including lack of granular MICs, use of surrogate intravenous antibiotics to infer susceptibility of oral agents, and lack of systemic susceptibility breakpoints for oral BLs. All of these limitations should be considered when designing future studies.

In conclusion, our data suggest that oral FQs and TMP-SMX are similar in effectiveness to IVBL therapy for GN-BSI/cUTI and may be considered for oral therapy transitions when the isolate is susceptible. TMP-SMX effectiveness might be optimized with total durations longer than 10 days, although further study is needed. Conversely, HBBL stepdown therapy was associated with higher recurrence rates regardless of treatment duration. Further studies are needed to determine whether optimized dosing and/or extended treatment duration can mitigate the higher risk of recurrence observed with HBBLs.

## Supplementary Material

ofae193_Supplementary_Data

## Supplementary Data

Supplementary materials are available at *Open Forum Infectious Diseases* online. Consisting of data provided by the authors to benefit the reader, the posted materials are not copyedited and are the sole responsibility of the authors, so questions or comments should be addressed to the corresponding author.
