# Supplementary material for: Real-World Effectiveness of Intravenous and Oral Antibiotic Stepdown Strategies for Gram-Negative Complicated Urinary Tract Infection With Bacteremia
Source: Open Forum Infect Dis. 2024 Apr 4;11(4):ofae193. doi: 10.1093/ofid/ofae193 (PMC11045028; doi:10.1093/ofid/ofae193)
Supplement: ofae193_Supplementary_Data [file ofae193_supplementary_data.zip › Comp GNB Supplemental Material 12-01-23.docx]

**Supplemental Material**

**Table S1. Balance Table for the Propensity Score Model**

| Standardized effect size | Minimum p-value | Maximum Kolmogorov-Smirnov test statistic | Minimum Kolmogorov-Smirnov p-value | Stop Method |
| --- | --- | --- | --- | --- |
| 1.3437 | 0 | 0.4630 | 0.0000 | Unweighted |
| 0.5600 | 0 | 0.1930 | 0.1002 | ES Mean |
| 0.4683 | 0 | 0.1867 | 0.1003 | KS Mean* |

* Both stop methods performed similarly. KS Mean weights had slightly larger effective sample sizes, so KS Mean was selected.

**Figure S1. Propensity Score Box Plots to Assess Overlap**


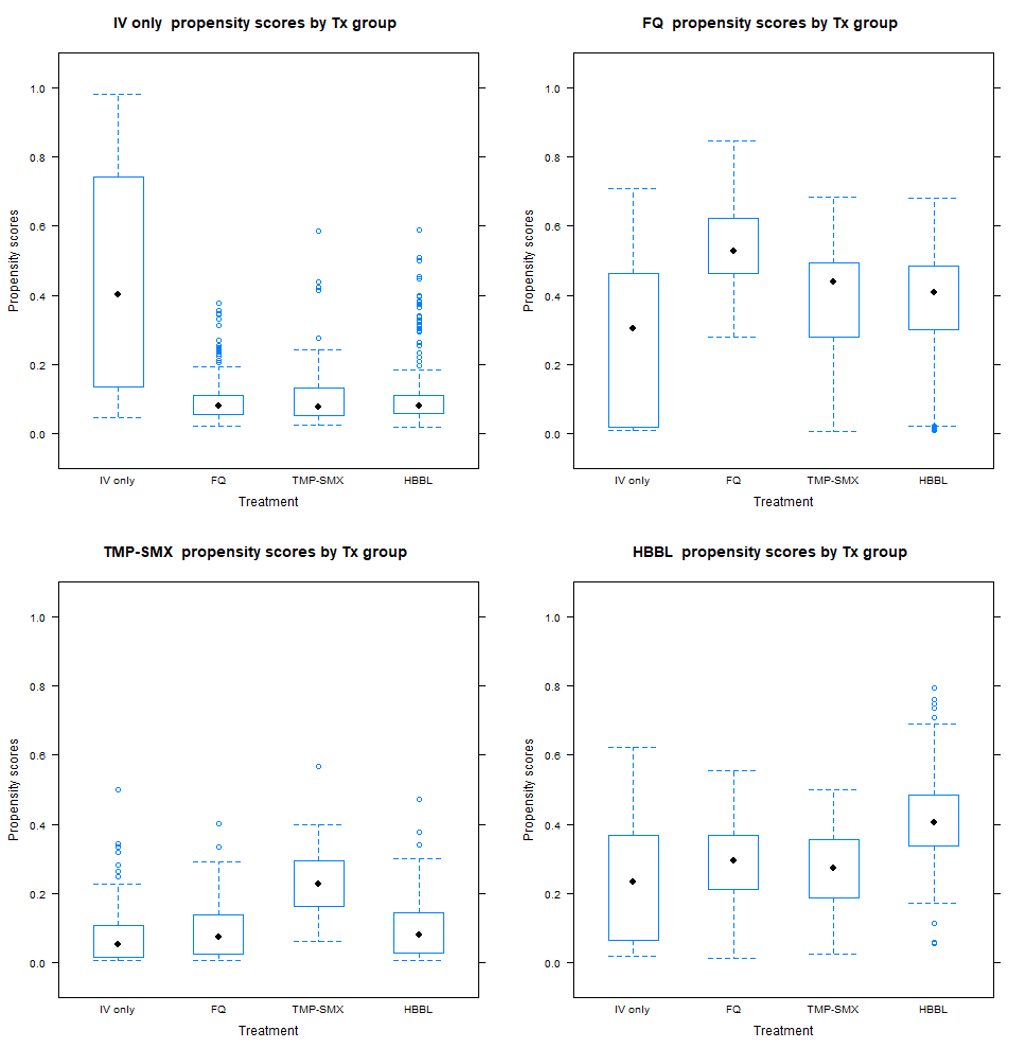


**Figure S2. Youden Index and Associated ROC Curve**


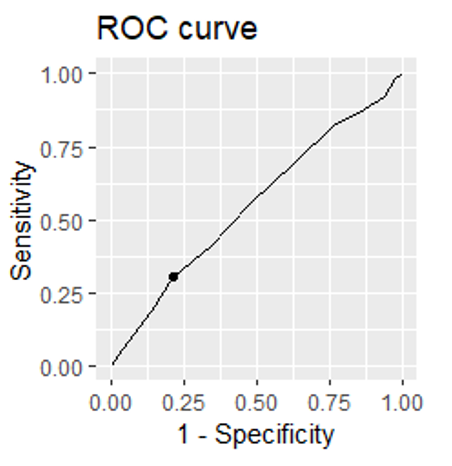

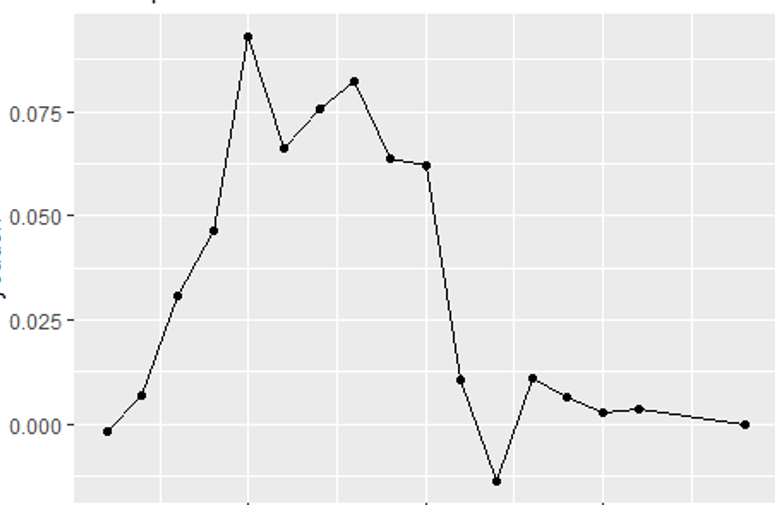


10-day Treatment Duration

**Youden Index**

6 7 8 9 10 11 12 13 14 15 16 17 18 19 20 21 22

**Total Treatment Duration (Days)**

**Figure S3. Recurrence-free days (through day 30) for complicated GN-BSI based on oral stepdown antibiotic**


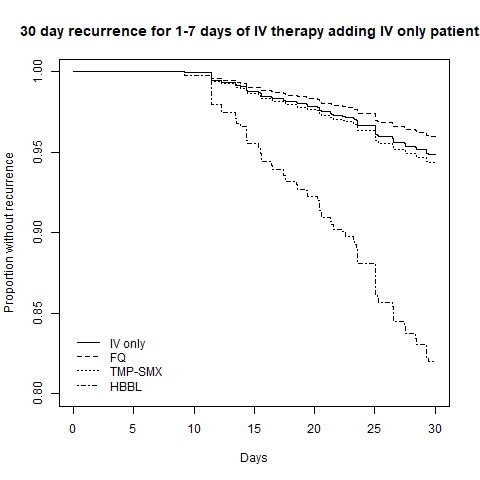


| **Number of**  **Patients** | **Variable** | **Hazard ratio (95% CI)** | **P-value** |
| --- | --- | --- | --- |
| 108 | IV β-lactams (IV only) – referent | - | - |
| 289 | Fluoroquinolones (FQ) | 0.77 (0.27, 2.20) | 0.630 |
| 73 | Trimethoprim-sulfamethoxazole (TMP-SMX) | 1.10 (0.30, 4.02) | 0.887 |
| 214 | High bioavailability β-lactams (HBBL) | **3.76 (1.41, 10.01)** | **0.008** |
| -- | Days of IV antibiotics | 1.12 (0.87, 1.43) | 0.385 |
| -- | Total days of IV and oral antibiotics | 0.93 (0.81, 1.07) | 0.333 |

**Figure S4. Recurrence-free days (through day 90) for complicated GN-BSI based on oral stepdown antibiotic**


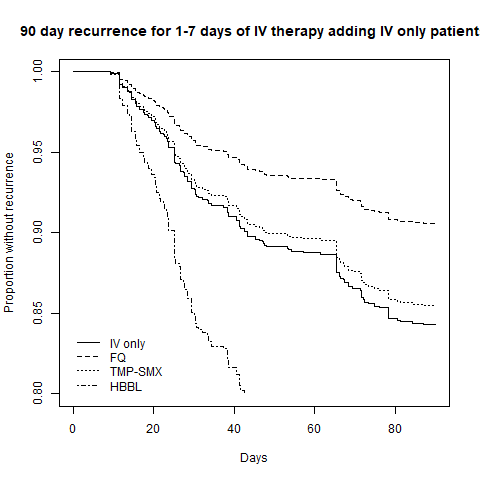


| **Number of**  **Patients** | **Variable** | **Hazard ratio (95% CI)** | **P-value** |
| --- | --- | --- | --- |
| 108 | IV β-lactams (IV only) – referent | - | - |
| 289 | Fluoroquinolones (FQ) | 0.58 (0.24, 1.41) | 0.229 |
| 73 | Trimethoprim-sulfamethoxazole (TMP-SMX) | 0.92 (0.36, 2.35) | 0.860 |
| 214 | High bioavailability β-lactams (HBBL) | 2.15 (0.95, 4.87) | 0.067 |
| -- | Days of IV antibiotics | 1.10 (0.89, 1.35) | 0.377 |
| -- | Total days of IV and oral antibiotics | 0.92 (0.84, 1.01) | 0.068 |


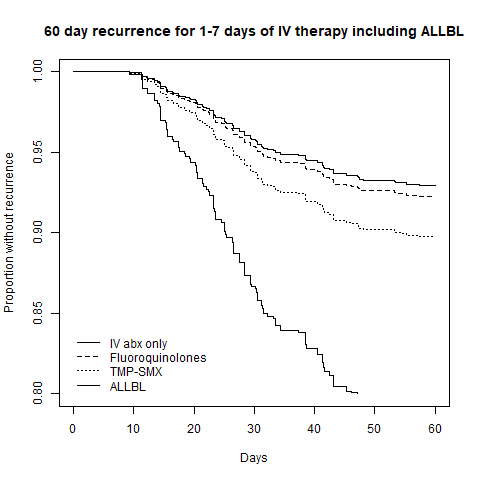
**Figure S5. Recurrence-free days (through day 60) for GN-BSI based on oral stepdown antibiotic (ALL beta-lactams)**

| **Number of**  **Patients** | **Variable** | **Hazard ratio (95% CI)** | **P-value** |
| --- | --- | --- | --- |
| 108 | IV β-lactams (IV only) – referent | - | - |
| 289 | Fluoroquinolones (FQ) | 1.10 (0.49, 2.48) | 0.810 |
| 73 | Trimethoprim-sulfamethoxazole (TMP-SMX) | 1.48 (0.55, 3.98) | 0.434 |
| 289 | All β-lactams (ALLBL):  HBBL (n = 214) + LBBL (n = 75) | **3.33 (1.65, 6.74)** | **<0.001** |
| -- | Days of IV antibiotics | 1.17 (0.97, 1.40) | 0.104 |
| -- | Total days of IV and oral antibiotics | **0.92 (0.85, 0.99)** | **0.034** |

**Figure S6. Recurrence-free days (through day 60) for GN-BSI based on oral stepdown antibiotic (low cefazolin MIC HBBLs)**

| **Number of**  **Patients** | **Variable** | **Hazard ratio (95% CI)** | **P-value** |
| --- | --- | --- | --- |
| 108 | IV β-lactams (IV only) – referent | - | - |
| 289 | Fluoroquinolones (FQ) | 1.05 (0.47, 2.33) | 0.905 |
| 73 | Trimethoprim-sulfamethoxazole (TMP-SMX) | 1.42 (0.53, 3.79) | 0.483 |
| 168 | HBBL and blood isolate was susceptible  at cefazolin MIC ≤ 2 mg/L (HBBL) | **3.53 (1.52, 8.21)** | **0.003** |
| -- | Days of IV antibiotics | 1.16 (0.91, 1.47) | 0.233 |
| -- | Total days of IV and oral antibiotics | 0.93 (0.83, 1.05) | 0.233 |


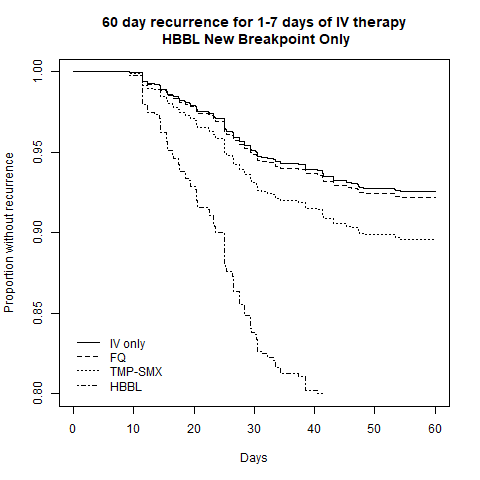


**Figure S7. Recurrence-free days (through day 60) for GN-BSI based on oral stepdown antibiotic**

**(restricted to 1-4 days IV therapy prior to discharge or oral stepdown)**

| **Number of**  **Patients** | **Variable** | **Hazard ratio (95% CI)** | **P-value** |
| --- | --- | --- | --- |
| 58 | IV β-lactams (IV only) – referent | - | - |
| 257 | Fluoroquinolones (FQ) | 0.99 (0.39, 2.51) | 0.988 |
| 50 | Trimethoprim-sulfamethoxazole (TMP-SMX) | 1.83 (0.55, 6.08) | 0.327 |
| 145 | High bioavailability β-lactams (HBBL) | **5.13 (2.04, 12.92)** | **<0.001** |
| -- | Days of IV antibiotics | **1.52 (1.03, 2.24)** | **0.035** |
| -- | Total days of IV and oral antibiotics | 0.95 (0.85, 1.07) | 0.409 |


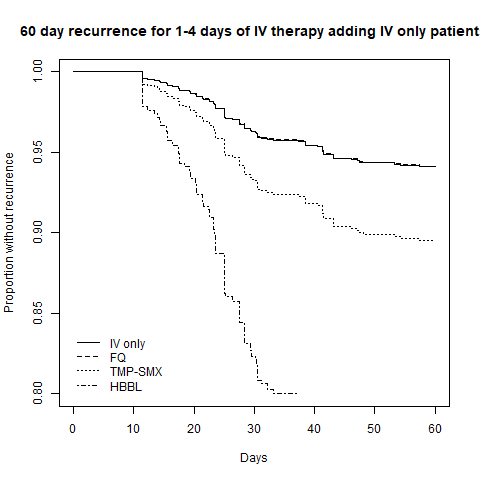


**Figure S8. Recurrence-free days (through day 60) for GN-BSI: HBBL, LBBL, or IVBL**

| **Number of**  **Patients** | **Variable** | **Hazard ratio (95% CI)** | **P-value** |
| --- | --- | --- | --- |
| 108 | IV β-lactams (IV only) – referent | **-** | **-** |
| 214 | High bioavailability β-lactams (HBBL) | **2.67 (1.28, 5.54)** | **0.009** |
| 75 | Low bioavailability β-lactams (LBBL) | **2.85 (1.28, 6.31)** | **0.010** |
| -- | Days of IV antibiotics | 0.96 (0.76, 1.22) | 0.744 |
| -- | Total days of IV and oral antibiotics | 1.00 (0.90, 1.11) | 0.980 |


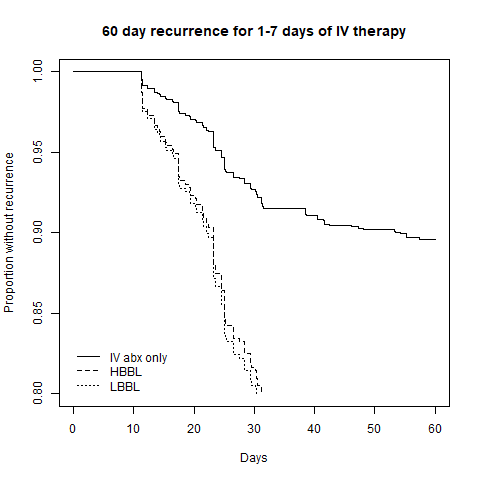


**Table S2. HBBL Stepdown Regimens and Unadjusted 60-day Recurrence for Complicated GN-BSI, Stratified by Renal Function**

| **Creatinine Clearance (mL/min) at Discharge** | **< 30**  **(n = 27)** | **30-50**  **(n = 65)** | **> 50**  **(n = 122)** |
| --- | --- | --- | --- |
| **Outcomes** |  |  |  |
| Recurrences at 60 days | 5 (18.5) | 14 (21.5) | 27 (22.1) |
| **Oral antibiotic dosing** |  |  |  |
| Amoxicillin |  |  |  |
| 500 – 1,000 mg q8h | 2 (7) | 6 (9) | 9 (7) |
| 500 – 1,000 mg q12h | 3 (11) | 1 (2) | 2 (2) |
| Amoxicillin-clavulanic acid |  |  |  |
| 500-875 mg q12h | 7 (26) | 15 (23) | 35 (29) |
| 500-875 mg q8h | -- | 1 (2) | 2 (2) |
| Cephalexin |  |  |  |
| 500 – 1,000 mg q6h | 1 (4) | 7 (11) | 52 (43) |
| 500 – 1,000 mg q8h | 11 (41) | 32 (49) | 22 (18) |
| 500 – 1,000 mg q12h | 3 (11) | 3 (5) | -- |

**Table S3. Oral Stepdown Regimens Stratified by Dosing and Unadjusted 60-day Recurrence**

| HBBL patients (n = 214) | **No Recurrence** | **Recurrence** | **P-value^1^** |
| --- | --- | --- | --- |
| **Received Recommended HBBL Dosing**  **(n = 93)** | 77 (83%) | 16 (17%) | 0.24 |
| **Received Lower HBBL Dosing**  **(n = 121)** | 91 (75%) | 30 (25%) |  |
|  |  |  |  |
| FQ patients (n = 289) | **No Recurrence** | **Recurrence** | **P-value^1^** |
| **Received Recommended FQ Dosing**  **(n = 199)** | 186 (93%) | 13 (7%) | 0.47 |
| **Lower FQ Dosing**  **(n = 90)** | 82 (91%) | 8 (9%) |  |
|  |  |  |  |
| TMP-SMX patients (n = 73) | **No Recurrence** | **Recurrence** | **P-value^1^** |
| **Received Recommended TMP-SMX Dosing**  **(n = 3)** | 2 (67%) | 1 (33%) | 0.26 |
| **Lower TMP-SMX Dosing**  **(n = 70)** | 64 (91%) | 6 (9%) |  |

^1^By Fisher’s exact test
